# Albuminuria during Pregnancy

**Published:** 1878-04

**Authors:** 


Albuminuria During Pregnancy.—C. H. Petit (*Centralbl. f. Med.*, 1877, p. 784, from *Annales de Gynecologie*) finds albuminuria a frequent condition during pregnancy, labor, and after delivery. Usually it appears during labor rather than in pregnancy. The albuminuria of labor is to be distinguished from that of pregnancy; young primiparæ are particularly disposed to it. The larger the child, the more abundant is the proportion of albumen in the urine. *Philadelphia Medical Times.*

